# National, State, and Local Area Vaccination Coverage Among Children Aged 19–35 Months — United States, 2012

**Published:** 2013-09-13

**Authors:** Carla L. Black, David Yankey, Maureen Kolasa

**Affiliations:** Immunization Services Div, National Center for Immunization and Respiratory Diseases, CDC

The National Immunization Survey (NIS) is a random-digit–dialed telephone survey used to monitor vaccination coverage among U.S. children aged 19–35 months. This report describes national, state, and selected local area vaccination coverage estimates for children born during January 2009–May 2011, based on results from the 2012 NIS. Healthy People 2020* objectives set childhood vaccination targets of 90% for ≥1 doses of measles, mumps, and rubella vaccine (MMR); ≥3 doses of hepatitis B vaccine (HepB); ≥3 doses of poliovirus vaccine; ≥1 doses of varicella vaccine; ≥4 doses of diphtheria, tetanus, and pertussis vaccine (DTaP); ≥4 doses of pneumococcal conjugate vaccine (PCV); and the full series of *Haemophilus influenzae* type b vaccine (Hib). Vaccination coverage remained near or above the national *Healthy People 2020* target for ≥1 doses of MMR (90.8%), ≥3 doses of poliovirus vaccine (92.8%), ≥3 doses of HepB (89.7%), and ≥1 doses of varicella vaccine (90.2%). Coverage increased from 68.6% in 2011 to 71.6% in 2012 for the birth dose of HepB.† Coverage was below the *Healthy People 2020* target and either decreased or remained stable relative to 2011 for ≥4 doses of DTaP (82.5%), the full series of Hib (80.9%), and ≥4 doses of PCV (81.9%). Coverage also remained stable relative to 2011 and below the *Healthy People 2020* targets of 85% and 80%, respectively, for ≥2 doses of hepatitis A vaccine (HepA) (53.0%), and rotavirus vaccine (68.6%). The percentage of children who had not received any vaccinations remained <1.0%. Although disparities in coverage were not observed for most racial/ethnic groups, children living in families with incomes below the federal poverty level had lower coverage than children living in families at or above the poverty level for ≥4 doses of DTaP (by 6.5 percentage points), the full Hib series (by 7.6 percentage points), ≥4 doses of PCV (by 8.6 percentage points), ≥2 doses of HepA (by 6.0 percentage points), and rotavirus vaccine (by 9.5 percentage points). Maintaining high coverage levels is important to maintain the current low burden of vaccine-preventable diseases in the United States and prevent their resurgence (1).

NIS uses a quarterly, random-digit–dialed sample of telephone numbers to reach households with children aged 19–35 months in the 50 states and selected local areas and territories,§ followed by a mail survey sent to the children’s vaccination providers to collect vaccination information. Data were weighted to represent the population of children aged 19–35 months, with adjustments for households with multiple telephone lines and mixed telephone use (landline and cellular), household nonresponse, and exclusion of households without telephone service.¶ Beginning in 2011, NIS changed from sampling only landline telephones to a dual-frame sampling scheme, with interviews conducted via landline or cellular telephone. The response rate** for the 2012 NIS was 64.7% for the landline telephone sample (including the U.S. Virgin Islands) and 30.6% for the cellular telephone sample. Providers returned vaccination records for 67.6% of the 12,727 children with completed household interviews from the landline sample and 63.9% of the 13,009 children with completed household interviews from the cellular telephone sample, for a total of 16,916 children with provider-reported vaccination records included in this report. Of this total, 8,313 (49%) were from the cellular telephone sample, of whom 5,281 were from households with only cellular telephone service. Because the number of Hib†† and rotavirus vaccine§§ doses required differs according to manufacturer, coverage estimates for these vaccines take into account the type of vaccine used. Logistic regression was used to examine differences among racial/ethnic groups, controlling for poverty status. Statistical analyses were conducted using t-tests based on weighted data and accounting for the complex survey design. A p-value of <0.05 was considered statistically significant.

In 2012, national vaccination coverage among children aged 19–35 months was 82.5% for ≥4 doses of DTaP, 92.8% for ≥3 doses of poliovirus vaccine, 90.8% for ≥1 doses of MMR, 89.7% for ≥3 doses of HepB, and 90.2% for ≥1 doses of varicella vaccine (Table 1). Although this represents a decline in coverage from 2011 of 1–2 percentage points for DTaP, poliovirus, and HepB, coverage for these vaccines has remained high and stable for at least the past decade.¶¶ Coverage with ≥4 doses of PCV decreased from 84.4% in 2011 to 81.9% in 2012. Coverage with the birth dose of HepB increased from 68.6% in 2011 to 71.6% in 2012. Coverage with the full series of Hib, which steadily increased during 2009–2011 after a vaccine shortage that occurred from December 2007 to September 2009 (2), was similar in 2012 at 80.9% compared with 2011. Similarly, coverage with ≥2 doses of HepA and rotavirus vaccine remained similar to 2011 levels at 53.0% and 68.6% in 2012, respectively. Coverage with the combined vaccine series (4:3:1:3*:3:1:4)*** was 68.4% in 2012, also similar to coverage in 2011.

Children in families with incomes below the federal poverty level††† had lower coverage than children in families at or above the poverty level for ≥3 and ≥4 doses of DTaP, primary and full series of Hib, ≥3 and ≥4 doses of PCV, ≥2 doses of HepA, rotavirus vaccine, and the combined vaccine series (Table 2). Children in families below the poverty level had higher HepB birth dose coverage than children living at or above the poverty level. No differences by poverty status were observed for poliovirus vaccine, MMR, ≥3 doses of HepB, or varicella vaccine.

Compared with white children,§§§ black children had lower coverage for ≥4 doses of DTaP, the full series of Hib, ≥4 doses of PCV, rotavirus vaccine, and the combined 4:3:1:3*:3:1:4 series (Table 2). After adjustment for poverty status, black race was not associated with coverage with any of these vaccines except for rotavirus. American Indian/Alaska Native (AI/AN) children and Asian children had higher coverage for ≥3 doses of HepB compared with white children. Black, Hispanic, and multiracial children had higher coverage for the birth dose of HepB compared with white children. With the exception of the difference in HepB birth dose coverage between Hispanic and white children, all of these associations with ≥3 doses of HepB and the birth dose of HepB remained statistically significant after adjustment for poverty status.

Vaccination coverage varied by state, with coverage for the combined vaccine series ranging from 59.5% in Alaska to 80.2% in Hawaii (Table 3). Fifteen states had point estimates of MMR coverage below the *Healthy People 2020* target of 90%, and only Connecticut, Delaware, and the District of Columbia had coverage ≥90% for ≥4 doses of DTaP. Variations in coverage were widest for the birth dose of HepB (ranging from 36.0% in Vermont to 87.6% in Georgia), ≥2 doses of HepA (ranging from 32.3% in Wyoming to 65.9% in Georgia), and rotavirus vaccine (ranging from 54.2% in the District of Columbia to 83.0% in New Hampshire).

## Editorial Note

The results of the 2012 NIS indicate that vaccination coverage among children aged 19–35 months continues to be near or above the *Healthy People 2020* target of 90% for MMR, poliovirus vaccine, HepB, and varicella vaccine. Although coverage estimates for many vaccines had small but statistically significant decreases compared with 2011, estimates are not directly comparable between years because NIS methods were changed. The number of interviews conducted via cellular telephone increased in 2012, such that approximately half of the 2012 NIS unweighted sample came from the cellular telephone sampling frame, compared with 11% of the 2011 unweighted sample. In 2012, an estimated 45% of U.S. children aged <18 years lived in households with cellular telephones only (3). The proportion of children aged 19–35 months living in households with only cellular telephone service estimated from the weighted 2012 NIS sample was 52.7%. Thus, the NIS sample now more closely resembles the U.S. population with respect to telephone service, and these 2012 vaccination coverage estimates should be considered a baseline against which subsequent trends in coverage can be evaluated.

After a sustained increase from 2009 to 2011, likely attributable to recovery from a Hib vaccine shortage that occurred from December 2007 to June 2009 (2), coverage with the full series of Hib vaccine has reached levels in 2012 similar to those of DTaP and PCV, vaccines that also require a booster dose during the second year of life. Because the frequency of recommended well-child visits declines after age 12 months, fewer opportunities for catch-up doses with these vaccines exist when children fall behind schedule. CDC encourages the use of provider and system-based interventions aimed at encouraging adherence to well-child visits and facilitating delivery of vaccines at these visits. Examples include use of immunization information systems, provider assessment and feedback, provider reminders, standing orders, and provider education in conjunction with other interventions (4).

Coverage with HepA and rotavirus, the more recently recommended vaccines, also remained similar in 2012 compared with 2011, after several years of continued increase. Similar to Hib, DTaP, and PCV, the plateau in coverage for HepA might be attributable to fewer opportunities for catch-up doses, as the first dose of HepA is recommended during age 12–23 months. Children’s vaccination status in NIS is determined up to age 19–35 months, so some children might have received their second dose, or might be due for the second dose, after the survey was conducted (the second dose is recommended 6–18 months after the first dose) (5). For rotavirus vaccine, the first dose should be given before age 14 weeks and 6 days because of insufficient evidence of safety in children aged >15 weeks, and the final dose should be given by age 8 months (5). These age restrictions might preclude infants from starting or completing the series. Health-care providers should make every effort to start and complete administration of the rotavirus vaccine series on time.

What is already known on this topic?

*Healthy People 2020* set childhood vaccination targets of 90% for ≥1 doses of measles, mumps, rubella vaccine (MMR); ≥3 doses of hepatitis B vaccine (HepB); ≥3 doses of poliovirus vaccine; ≥1 doses of varicella vaccine; ≥4 doses of diphtheria, tetanus, and pertussis vaccine; ≥4 doses of pneumococcal conjugate vaccine; and the full series of *Haemophilus influenzae* type b vaccine. The National Immunization Survey estimates coverage among U.S. children aged 19–35 months for these and other vaccines.

What is added by this report?

In 2012, childhood vaccination coverage remains near or above national target levels for ≥1 doses of MMR (90.8%), ≥3 doses of HepB (89.7%), ≥3 doses of poliovirus vaccine (92.8%), and ≥1 doses of varicella vaccine (90.2%); however, coverage varied by state and tended to be lower among children in families with incomes below the federal poverty level.

What are the implications for public health practice?

Sustaining current coverage levels and increasing coverage for those vaccines below national target levels is needed to maintain the low levels of vaccine-preventable diseases and prevent a resurgence of these diseases in the United States. Ensuring systems such as client reminder/recall and vaccination programs are in place in settings such as Special Supplemental Nutrition Program for Women, Infants, and Children (WIC) clinics and child-care facilities can help support high vaccination coverage.

Although few differences in coverage by racial/ethnic group were observed after adjustment for poverty status, differences in coverage by poverty level remained for many vaccines. The Vaccines For Children program¶¶¶ has been successful in removing differences in coverage between children living above and below the poverty level that once existed for vaccines such as MMR, polio, and HepB (6); however, coverage among children living below the poverty level still lags behind coverage of children living at or above the poverty level for newer vaccines (HepA and rotavirus) and vaccines that require 4 doses to complete the series.

Vaccination coverage continues to vary across states. Clusters of unvaccinated children leave communities vulnerable to outbreaks of disease. The continued occurrence of measles outbreaks among unvaccinated persons in the United States (7) underscores the importance of maintaining uniformly high coverage to prevent transmission of imported disease. Recent budget cuts to state and local health departments (8) as well as differences by state in factors such as population characteristics, immunization program activities, vaccination requirements for child-care centers, and vaccine financing policies might contribute to variations in vaccination coverage.

The findings in this report are subject to at least four limitations. First, the proportion of the NIS sampled by cellular telephone in 2012 was about half compared with only 11% in 2011 and zero in earlier years. Living in a household with only cellular telephone service is associated with poverty and other demographic factors that might be related to vaccination status (3). Second, underestimates of vaccination coverage might have resulted from the exclusive use of provider-reported vaccination histories because completeness of these records is unknown. Third, bias resulting from nonresponse and exclusion of households without telephone service might persist after weighting adjustments, although estimated bias from these sources for the 2011 NIS was low for selected vaccines examined, ranging from 0.3 (for MMR) to 1.5 (for ≥4 DTaP) percentage points (9). The potential for nonresponse bias was increased in 2012 because of the lower response rate for the cellular telephone sample. However, a comparison of vaccination coverage estimates from the NIS from July 2011 through June 2012 with those from the National Health Interview Survey during the same period yielded similar results, both overall and for children living in cellular-only households, despite largely different response rates between the two surveys (Assessment Branch, Immunization Services Division, National Center for Immunization and Respiratory Diseases, and Survey Planning and Special Surveys Branch, Division of Health Interview Statistics, National Center for Health Statistics, CDC; unpublished data; 2013). Finally, although national coverage estimates are precise, estimates for state and local areas should be interpreted with caution because of smaller sample sizes and wider confidence intervals.

High vaccination coverage among preschool-aged children has resulted in historically low levels of most vaccine-preventable diseases in the United States (1). The results of the 2012 NIS indicate that vaccination coverage among young children remained relatively stable and the proportion of children who do not receive any vaccinations has remained low. Slight decreases in coverage for some vaccines relative to 2011 cannot be immediately explained but could be attributable to a change in NIS methods. The 2012 results should be considered a baseline against which future trends in coverage can be evaluated. Careful monitoring of coverage levels overall and in subpopulations (e.g., racial/ethnic and geographic) is important to ensure that all children remain adequately protected. Parents and health-care providers should work to sustain high coverage and improve coverage for the more recently recommended vaccines and those that require booster doses after age 12 months. In addition to health system–based interventions previously described, national, state and local immunization programs should continue to partner with providers to implement the *Guide to Community Preventive Services*–recommended interventions aimed at increasing community demand for vaccination, such as client reminder/recall and client or family incentives. Enhanced access to health services also is recommended, through reduced out-of-pocket costs, home visits, and vaccination programs in child-care centers, schools, and Special Supplemental Nutrition Program for Women, Infants, and Children (WIC) settings**** (4). Health insurance reforms of the Affordable Care Act require health plans to cover recommended immunizations without cost to the enrollee when administered by an in-network provider (10).††††

## Figures and Tables

**TABLE 1:** Estimated vaccination coverage among children aged 19–35 months, by selected vaccines and dosages — National Immunization Survey, United States, 2008–2012*

	2008		2009		2010		2011		2012	
					
Vaccine and dosage	%	(95% CI)	%	(95% CI)	%	(95% CI)	%	(95% CI)	%	(95% CI)
**DTaP**										
≥3 doses	96.2	(±0.5)	95.0	(±0.6)	95.0	(±0.6)	95.5	(±0.5)	94.3	(±0.7)†
≥4 doses	84.6	(±1.0)	83.9	(±1.0)	84.4	(±1.0)	84.6	(±1.0)	82.5	(±1.2)†
**Poliovirus (≥3 doses)**	93.6	(±0.6)	92.8	(±0.7)	93.3	(±0.7)	93.9	(±0.6)	92.8	(±0.7)†
**MMR (≥1 doses)**	92.1	(±0.7)	90.0	(±0.8)	91.5	(±0.7)	91.6	(±0.8)	90.8	(±0.8)
**Hib** §										
Primary series	N/A		92.1	(±0.8)	92.2	(±0.8)	94.2	(±0.6)	93.3	(±0.7)
Full series	N/A		54.8	(±1.4)	66.8	(±1.3)	80.4	(±1.1)	80.9	(±1.2)
**HepB**										
≥3 doses	93.5	(±0.7)	92.4	(±0.7)	91.8	(±0.7)	91.1	(±0.7)	89.7	(±0.9)†
1 dose by 3 days (birth)¶	55.3	(±1.3)	60.8	(±1.3)	64.1	(±1.3)	68.6	(±1.3)	71.6	(±1.4)†
**Varicella (≥1 doses)**	90.7	(±0.7)	89.6	(±0.8)	90.4	(±0.8)	90.8	(±0.7)	90.2	(±0.8)
**PCV**										
≥3 doses	92.8	(±0.6)	92.6	(±0.7)	92.6	(±0.8)	93.6	(±0.6)	92.3	(±0.8)†
≥4 doses	80.1	(±1.1)	80.4	(±1.2)	83.3	(±1.0)	84.4	(±1.0)	81.9	(±1.1)†
**HepA** **										
≥1 doses	70.5	(±1.1)	75.0	(±1.1)	78.3	(±1.1)	81.2	(±1.0)	81.5	(±1.1)
≥2 doses	40.4	(±1.2)	46.6	(±1.4)	49.7	(±1.4)	52.2	(±1.4)	53.0	(±1.5)
**Rotavirus** ††	N/A		43.9	(±1.4)	59.2	(±1.4)	67.3	(±1.3)	68.6	(±1.4)
**Combined series**										
4:3:1:3*:3:1:4§§	N/A		44.3	(±1.4)	56.6	(±1.3)	68.5	(±1.3)	68.4	(±1.4)
**Children who received no vaccinations**	0.6	(±0.2)	0.6	(±0.1)	0.7	(±0.2)	0.8	(±0.2)	0.8	(±0.1)

**Abbreviations:** CI = confidence interval; DTaP = diphtheria and tetanus toxoids and acellular pertussis vaccine (includes children who might have been vaccinated with diphtheria and tetanus toxoids and pertussis vaccine or diphtheria and tetanus toxoids vaccine); MMR = measles, mumps, and rubella vaccine; Hib = *Haemophilus influenzae* type b vaccine; N/A = not available (estimate not available if the unweighted sample size for the denominator was <30 or 95% CI half width / estimate >0.588 or 95% CI half width >10); HepB = hepatitis B vaccine; HepA = hepatitis A vaccine; PCV = pneumococcal conjugate vaccine.

* For 2008, includes children born during January 2005–June 2007; for 2009, children born during January 2006–July 2008; for 2010, children born during January 2007–July 2009; for 2011, children born during January 2008–May 2010; and for 2012, children born during January 2009–May 2011.

† Statistically significant change in coverage compared with 2011 (p<0.05).

§ Hib primary series: receipt of ≥2 or ≥3 doses, depending on product received. Full series: receipt of ≥3 or ≥4 doses, depending on product received (primary series and booster dose). Hib coverage for primary or full series not available until 2009.

¶ HepB administered from birth through age 3 days.

** HepA coverage not available before 2008.

†† Rotavirus vaccine includes ≥2 or ≥3 doses, depending on the product received (≥2 doses for Rotarix [RV1] or ≥3 doses for RotaTeq [RV5]). Estimates of rotavirus vaccine coverage not available before 2009.

§§ 4:3:1:3*:3:1:4 series, referred to as routine, includes ≥4 doses of DTaP, ≥3 doses of poliovirus vaccine, ≥1 doses of measles vaccine, full series of Hib (3 or 4 doses, depending on product), ≥3 doses of HepB, ≥1 doses of varicella vaccine, and ≥4 doses of PCV.

**TABLE 2:** Estimated vaccination coverage among children aged 19–35 months, by selected vaccines and dosages, race/ethnicity,* and poverty level† — National Immunization Survey, United States, 2012§

	Race/Ethnicity												Poverty level			
		
	White, non-Hispanic		Black, non-Hispanic		Hispanic		American Indian/Alaska Native		Asian		Multiracial, non-Hispanic		At or above		Below	
								
Vaccine and dosage	%	(95% CI)	%	(95% CI)	%	(95% CI)	%	(95% CI)	%	(95% CI)	%	(95% CI)	%	(95% CI)	%	(95% CI)
**DTaP**																
≥3 doses	94.8	(±0.8)	94.0	(±1.6)	93.5	(±1.9)	95.6	(±3.3)	96.1	(±2.1)	95.1	(±2.0)	95.0	(±0.9)	93.4	(±1.3)**
≥4 doses	83.6	(±1.5)	79.6	(±3.1)¶	80.8	(±2.9)	88.2	(±5.9)	88.1	(±4.3)	85.6	(±3.6)	85.0	(±1.4)	78.5	(±2.3)**
**Poliovirus (≥3 doses)**	93.0	(±0.9)	92.9	(±1.8)	92.5	(±1.8)	95.2	(±3.4)	92.3	(±3.6)	93.3	(±2.3)	93.4	(±0.9)	91.8	(±1.4)
**MMR (≥1 doses)**	90.9	(±1.0)	90.9	(±2.1)	90.7	(±2.0)	92.0	(±5.0)	89.8	(±5.2)	92.3	(±2.6)	91.4	(±1.0)	89.9	(±1.6)
**Hib** ††																
Primary series	93.7	(±0.9)	91.1	(±2.2)	93.5	(±1.7)	94.5	(±3.9)	94.9	(±2.2)	94.0	(±2.2)	94.3	(±0.8)	91.9	(±1.4)**
Full series	82.2	(±1.4)	77.5	(±3.3)¶	79.5	(±2.8)	84.7	(±7.1)	86.1	(±4.4)	82.5	(±3.9)	84.0	(±1.4)	76.4	(±2.2)**
**HepB**																
≥3 doses	89.3	(±1.1)	89.7	(±2.2)	89.4	(±2.1)	94.0	(±3.9)¶	93.2	(±2.7)¶	92.2	(±2.6)	89.8	(±1.1)	89.4	(±1.5)
1 dose by 3 days (birth)§§	69.2	(±1.6)	74.9	(±3.6)¶	73.9	(±3.4)¶	NA		71.6	(±6.6)	75.9	(±4.8)¶	69.4	(±1.7)	75.8	(±2.5)**
**Varicella (≥1 doses)**	89.8	(±1.0)	90.4	(±2.1)	90.9	(±2.1)	92.5	(±4.5)	91.9	(±3.2)	90.9	(±2.9)	90.6	(±1.0)	89.7	(±1.7)
**PCV**																
≥3 doses	92.7	(±1.0)	91.2	(±2.0)	92.4	(±1.8)	94.0	(±4.0)	90.7	(±3.3)	94.0	(±2.2)	93.4	(±0.9)	90.7	(±1.5)**
≥4 doses	83.5	(±1.4)	77.1	(±3.5)¶	82.1	(±2.5)	NA		80.7	(±5.1)	84.1	(±3.7)	85.3	(±1.2)	76.7	(±2.3)**
**HepA (≥2 doses)**	52.6	(±1.8)	52.0	(±3.9)	54.4	(±3.4)	NA		57.5	(±7.7)	49.4	(±5.7)	55.4	(±1.8)	49.4	(±2.7)**
**Rotavirus** ¶¶	70.5	(±1.6)	60.4	(±4.0)¶	70.0	(±3.1)	NA		69.9	(±7.1)	69.3	(±5.4)	72.5	(±1.6)	63.0	(±2.5)**
**Combined series**																
4:3:1:3*:3:1:4***	69.3	(±1.7)	64.8	(±3.8)¶	67.8	(±3.2)	NA		71.6	(±6.6)	71.5	(±4.8)	71.6	(±1.6)	63.4	(±2.7)**

**Abbreviations:** CI = confidence interval; DTaP = diphtheria and tetanus toxoids and acellular pertussis vaccine (includes children who might have been vaccinated with diphtheria and tetanus toxoids and pertussis vaccine or diphtheria and tetanus toxoids vaccine); Hib = *Haemophilus influenzae* type b vaccine; MMR = measles, mumps, and rubella vaccine; N/A = not available (estimate not available if the unweighted sample size for the denominator was <30 or 95% CI half width / estimate >0.588 or 95% CI half width >10); HepB = hepatitis B vaccine; HepA = hepatitis A vaccine; PCV = pneumococcal conjugate vaccine.

* Native Hawaiian or other Pacific Islanders not included because of small sample sizes.

† Poverty level was determined for all children. Children were classified as below poverty if their total family income was less than the poverty threshold specified for the applicable family size and number of children aged <18 years. All others were classified as at or above poverty. Poverty thresholds reflect yearly changes in the Consumer Price Index. Thresholds and guidelines available at http://www.census.gov/hhes/www/poverty.html.

§ Children in the 2012 National Immunization Survey were born during January 2009–May 2011.

¶ Estimates are statistically significant at p<0.05. Children identified as non-Hispanic white were the reference group.

** Estimates are statistically significant at p<0.05. Children living at or above poverty were the reference group.

†† Hib primary series: receipt of ≥2 or ≥3 doses, depending on product received; full series: primary series and booster dose includes receipt of ≥3 or ≥4 doses, depending on product received.

§§ HepB (≥1 doses) administered from birth through age 3 days.

¶¶ Includes ≥2 or ≥3 doses, depending on product received (≥2 doses for Rotarix [RV1] or ≥3 doses for RotaTeq [RV5]).

*** 4:3:1:3:3:1:4 series includes ≥4 doses of DTaP, ≥3 doses of poliovirus vaccine, ≥1 doses of measles vaccine, full series of Hib (3 or 4 doses, depending on type), ≥3 doses of HepB, ≥1 doses of varicella vaccine, and ≥4 doses of PCV.

**TABLE 3:** Estimated vaccination coverage among children aged 19–35 months, by selected individual vaccines and vaccination series* and state/local area — National Immunization Survey, United States, 2012†

	MMR (≥1 doses)		DTaP (≥4 doses)		HepB (birth)§		HepA (≥2 doses)¶		Rotavirus**		Combined series*	
						
State/Area	%	(95% CI)	%	(95% CI)	%	(95% CI)	%	(95% CI)	%	(95% CI)	%	(95% CI)
**United States**	**90.8**	**(±0.8)**	**82.5**	**(±1.2)** §§	**71.6**	**(±1.4)** ††	**53.0**	**(±1.5)**	**68.6**	**(±1.4)**	**68.4**	**(±1.4)**
Alabama	93.1	(±3.5)	84.8	(±5.9)	83.8	(±4.9)††	49.2	(±7.4)	66.0	(±7.4)§§	71.3	(±6.8)
Alaska	86.2	(±5.1)	79.4	(±5.8)	56.8	(±6.9)	50.1	(±6.9)	60.3	(±6.8)	59.5	(±6.8)
Arizona	88.3	(±4.9)	82.7	(±5.8)	83.0	(±5.3)††	55.2	(±6.9)	71.6	(±6.7)	67.5	(±7.5)
Arkansas	92.3	(±4.0)	79.8	(±6.4)	81.7	(±6.5)	40.1	(±7.5)	56.3	(±8.0)	66.4	(±7.6)
California	91.5	(±4.3)	81.6	(±6.6)	61.5	(±7.5)	54.6	(±7.8)	71.0	(±6.8)	66.8	(±7.5)
Colorado	91.5	(±4.5)	82.8	(±6.7)	64.0	(±8.4)	56.2	(±8.6)	73.5	(±7.7)	71.7	(±7.9)
Connecticut	94.8	(±2.9)	91.3	(±3.8)	75.7	(±5.7)	65.5	(±6.3)††	72.5	(±6.4)	77.1	(±5.7)
Delaware	94.4	(±3.4)	90.9	(±4.3)	72.3	(±6.7)	65.7	(±7.1)††	76.5	(±6.5)	72.6	(±6.7)
District of Columbia	93.0	(±3.7)	90.7	(±4.0)	78.2	(±5.4)	62.3	(±6.6)	54.2	(±6.8)	73.4	(±6.2)
Florida	91.0	(±4.8)	83.3	(±6.5)	62.6	(±7.6)	51.9	(±8.1)	66.0	(±7.9)	68.6	(±7.5)
Georgia	91.9	(±4.2)	86.7	(±5.2)	87.6	(±5.1)	65.9	(±7.6)	71.8	(±7.2)	74.7	(±6.8)
Hawaii	95.0	(±2.7)	87.9	(±4.6)	82.7	(±5.2)††	58.1	(±7.1)	70.6	(±6.5)††	80.2	(±5.5)
Idaho	93.3	(±3.6)	76.6	(±6.7)	70.1	(±7.8)	52.8	(±8.6)	68.2	(±7.2)	63.0	(±8.2)
Illinois	91.6	(±2.7)	85.3	(±2.6)	71.3	(±5.0)	48.2	(±5.4 )	67.2	(±5.2)	68.5	(±4.9)
City of Chicago	86.8	(±6.1)	79.4	(±7.6)	70.3	(±8.4)	45.2	(±8.7)	69.5	(±8.7)	60.4	(±8.8)§§
Rest of state	93.2	(±2.9)	87.4	(±4.1)	71.7	(±6.0)	49.3	(±6.6)	66.4	(±6.3)	71.4	(±5.8)
Indiana	90.0	(±4.5)	76.8	(±6.5)	78.2	(±6.0)	48.0	(±7.5)	63.9	(±7.4)	61.4	(±7.4)
Iowa	93.3	(±3.4)††	88.2	(±4.4)	68.3	(±7.5)	59.3	(±7.2)††	70.2	(±7.5)	74.8	(±6.3)
Kansas	88.5	(±4.6)	79.0	(±6.0)§§	78.3	(±5.4)	58.5	(±6.9)	59.9	(±7.0)	65.0	(±6.7)
Kentucky	89.2	(±4.4)	83.0	(±5.4)	80.8	(±5.6)	48.4	(±7.0)	69.0	(±6.4)	68.2	(±6.6)
Louisiana	90.5	(±4.0)	77.8	(±6.6)	76.6	(±6.8)	46.9	(±7.3)	65.0	(±7.4)	68.5	(±7.1)
Maine	91.2	(±4.2)	87.9	(±5.1)	74.2	(±5.8)	52.5	(±7.4)††	64.7	(±7.0)	72.6	(±6.6)
Maryland	92.5	(±4.8)	83.2	(±6.2)	73.3	(±6.6)	53.1	(±7.3)	71.2	(±6.9)	67.1	(±7.1)
Massachusetts	93.7	(±3.4)	88.2	(±4.5)	74.0	(±6.2)	57.5	(±6.9)	82.4	(±5.6)	73.5	(±6.2)
Michigan	91.4	(±4.4)	81.5	(±6.7)	78.9	(±6.1)	40.9	(±7.4)§§	64.3	(±7.4)	70.5	(±7.3)
Minnesota	90.1	(±5.6)	84.2	(±5.6)	62.8	(±7.4)	55.4	(±7.7)	76.6	(±6.4)	66.2	(±7.6)
Mississippi	93.4	(±4.3)	83.6	(±6.4)	81.6	(±6.5)	39.7	(±8.2)	63.8	(±8.0)	77.5	(±7.0)
Missouri	92.7	(±4.1)	81.9	(±7.0)	78.7	(±6.2)	56.3	(±7.9)	69.3	(±7.8)	63.9	(±8.0)
Montana	91.5	(±4.0)	86.6	(±4.4)††	64.5	(±6.8)§§	50.5	(±7.3)	61.3	(±7.4)	66.5	(±7.1)
Nebraska	89.0	(±4.4)§§	84.5	(±5.2)§§	79.4	(±5.8)	60.6	(±7.0)	74.2	(±6.2)	72.6	(±6.5)
Nevada	89.8	(±4.1)	81.0	(±5.5)	70.5	(±6.3)	52.2	(±7.0)	62.7	(±6.7)	65.3	(±6.6)
New Hampshire	93.7	(±3.4)	88.7	(±4.7)	72.2	(±6.6)	57.0	(±7.0)	83.0	(±5.8)	80.1	(±5.7)††
New Jersey	94.8	(±2.7)	84.7	(±5.1)	52.6	(±6.9)	45.9	(±6.9)	68.0	(±6.6)††	71.5	(±6.4)
New Mexico	88.8	(±4.4)	87.0	(±4.9)	68.9	(±7.0)	51.9	(±7.6)	78.4	(±5.8)	71.6	(±6.6)
New York	90.2	(±2.9)	83.8	(±3.5)	61.5	(±4.7)††	45.9	(±4.7)	65.5	(±4.5)	63.7	(±4.6)
City of New York	90.3	(±3.9)	82.9	(±5.3)	60.5	(±6.4)††	44.4	(±6.6)	56.8	(±6.8)	62.8	(±6.5)
Rest of state	90.0	(±4.2)	84.6	(±4.7)	62.4	(±6.8)	47.5	(±6.7)	74.1	(±5.9)††	64.6	(±6.5)
North Carolina	89.0	(±4.9)	85.9	(±5.4)	78.2	(±5.9)	48.5	(±7.3)	68.0	(±7.1)	75.4	(±6.5)
North Dakota	90.6	(±5.4)	85.1	(±6.2)	82.3	(±5.6)	59.8	(±7.5)	75.4	(±7.1)	72.2	(±7.2)
Ohio	90.3	(±4.9)	83.3	(±6.0)	77.8	(±6.2)	53.8	(±7.0)	67.4	(±7.5)	66.8	(±6.9)
Oklahoma	90.0	(±4.8)	79.1	(±6.0)	67.4	(±7.4)	56.1	(±7.4)	56.4	(±7.7)	61.0	(±7.6)
Oregon	87.3	(±4.7)	81.2	(±5.8)	65.4	(±6.6)	57.6	(±7.0)	66.1	(±6.7)	66.7	(±6.7)
Pennsylvania	87.0	(±4.6)§§	80.1	(±5.3)	83.2	(±4.3)††	58.5	(±6.1)	72.5	(±5.5)	68.3	(±5.9)
Philadelphia County	92.6	(±4.3)	85.4	(±5.7)	78.1	(±6.0)	58.1	(±7.6)	68.0	(±7.2)	73.8	(±7.1)
Rest of state	85.9	(±5.5)§§	79.1	(±6.2)	84.2	(±5.1)††	58.6	(±7.1)	73.4	(±6.4)	67.2	(±6.9)
Rhode Island	94.3	(±3.1)	89.0	(±4.9)	68.3	(±6.7)	57.3	(±6.9)	79.8	(±6.4)	72.5	(±6.5)
South Carolina	93.2	(±3.5)	80.9	(±6.0)	78.4	(±5.8)††	48.5	(±7.3)	70.6	(±6.7)††	71.8	(±6.7)
South Dakota	93.3	(±3.0)	79.2	(±5.5)	76.6	(±5.6)	45.3	(±6.8)††	59.5	(±7.0)	63.6	(±6.4)
Tennessee	92.2	(±4.0)	82.0	(±6.0)	68.8	(±7.0)	55.4	(±7.7)	64.3	(±7.6)	73.1	(±6.8)
Texas	89.7	(±2.4)§§	77.4	(±3.6)§§	74.6	(±3.7)	57.4	(±4.0)	67.5	(±3.9)	64.8	(±4.0)§§
Bexar County	90.9	(±4.0)	77.5	(±6.4)	76.4	(±6.4)††	62.6	(±7.6)	67.5	(±7.4)	65.7	(±7.5)
City of Houston	92.2	(±4.7)	83.4	(±6.8)	84.3	(±5.6)	64.4	(±8.4)	79.7	(±7.6)††	70.9	(±7.9)
Dallas County	86.5	(±5.6)	78.8	(±6.6)	72.3	(±7.0)§§	56.8	(±8.0)	72.0	(±7.2)	69.8	(±7.5)
El Paso County	87.1	(±4.7)	76.5	(±6.1)	77.9	(±5.6)	57.4	(±6.7)	68.4	(±6.7)	62.3	(±6.7)
Rest of state	89.7	(±3.3)§§	76.2	(±5.0)	72.8	(±5.2)	55.7	(±5.6)	64.5	(±5.4)§§	62.9	(±5.6)§§
Utah	87.3	(±5.5)	80.5	(±6.6)	78.6	(±6.3)	57.1	(±7.7)	74.5	(±6.8)	73.0	(±7.2)
Vermont	91.7	(±3.8)	86.0	(±5.0)	36.0	(±6.7)††	37.4	(±6.4)	64.2	(±6.6)	63.2	(±6.7)
Virginia	94.3	(±3.9)	82.7	(±6.6)	71.4	(±7.4)	50.0	(±8.3)	71.9	(±7.9)	69.8	(±7.7)
Washington	84.8	(±5.8)	84.0	(±5.5)	73.2	(±6.5)	51.0	(±7.4)	68.6	(±7.0)	65.2	(±7.2)
West Virginia	84.6	(±6.0)	79.1	(±6.8)	74.4	(±6.6)††	54.9	(±7.9)	62.6	(±7.8)	60.8	(±7.9)
Wisconsin	89.3	(±5.2)	87.8	(±5.3)	72.2	(±6.5)	55.6	(±7.4)	67.4	(±7.1)	75.2	(±6.5)
Wyoming	91.2	(±3.9)	79.4	(±6.0)	64.8	(±7.1)	32.3	(±6.8) §§	69.1	(±6.7)††	67.2	(±6.8)
U.S. Virgin Islands	63.7	(±7.4)§§	55.6	(±7.7)	72.8	(±7.0)	12.0	(±4.7)	15.6	(±5.7)	41.5	(±7.6)

**Abbreviations:** CI = confidence interval; DTaP = diphtheria and tetanus toxoids and acellular pertussis vaccine (includes children who might have been vaccinated with diphtheria and tetanus toxoids and pertussis vaccine or diphtheria and tetanus toxoids vaccine); HepB = hepatitis B vaccine; HepA = hepatitis A vaccine; Hib = *Haemophilus influenzae* type b vaccine; MMR = measles, mumps, and rubella vaccine; PCV = pneumococcal conjugate vaccine.

* Includes ≥4 doses of DTaP, ≥3 doses of poliovirus vaccine, ≥1 doses of measles vaccine, full series of Hib (3 or 4 doses, depending on product), ≥3 doses of HepB, ≥1 doses of varicella vaccine, and ≥4 doses of PCV.

† Children in the 2012 National Immunization Survey were born during January 2009–May 2011.

§ HepB administered from birth through age 3 days.

¶ ≥2 doses HepA and measured among children aged 19–35 months.

** ≥2 or ≥3 doses of rotavirus vaccine, depending on product received (≥2 doses for Rotarix [RV1] or ≥3 doses for RotaTeq [RV5]).

†† Statistically significant increase in coverage compared with 2011 (p<0.05).

§§ Statistically significant decrease in coverage compared with 2011 (p<0.05).

## Footnotes

* Additional information available at http://healthypeople.gov/2020/topicsobjectives2020/objectiveslist.aspx?topicid=23. The *Healthy People 2020* targets for children aged 19–35 months are 90%, except for rotavirus vaccine (80%) and ≥2 doses of HepA (85%).

† The *Healthy People 2020* target for the birth dose (0–3 days) of HepB is 85%, measured by annual birth cohort. In the two most recent complete birth cohorts captured by NIS, coverage with the birth dose of HepB was 65.0% for children born in 2008 and 70.6% for children born in 2009.

§ The eight local areas separately sampled for the 2012 NIS included six areas that receive federal Section 317 immunization funds and are included in the NIS sample every year (District of Columbia; Chicago, Illinois; New York, New York; Philadelphia County, Pennsylvania; Bexar County, Texas; and Houston, Texas) and two additional sampled areas (Dallas County, Texas, and El Paso County, Texas). The territory of the U.S. Virgin Islands (including St. Croix, St. Thomas, and St. John) was included in the 2012 NIS landline sample, but data from the U.S. Virgin Islands are excluded from national coverage estimates.

¶ A description of the statistical methodology of the NIS is available at http://www.cdc.gov/nchs/data/series/sr_02/sr02_138.pdf and ftp://ftp.cdc.gov/pub/health_statistics/nchs/dataset_documentation/nis/nispuf11_dug.pdf.

** The Council of American Survey Research Organization (CASRO) household response rate, calculated as the product of the resolution rate (percentage of the total telephone numbers called that were classified as nonworking, nonresidential, or residential), screening completion rate (percentage of known households that were successfully screened for the presence of age-eligible children), and the interview completion rate (percentage of households with one or more age-eligible children that completed the household survey). Additional information is available at http://casro.org. The CASRO response rate is equivalent to the American Association for Public Opinion Research (AAPOR) type 3 response rate. Information about AAPOR response rates is available at http://www.aapor.org/am/template.cfm?section=standard_definitions1&template=/cm/contentdisplay.cfm&contentid=1814.

†† Coverage for the primary Hib series was based on receipt of ≥2 or ≥3 doses, depending on product type received. The PRP-OMB Hib products require a 2-dose primary series with doses at ages 2 months and 4 months. All other Hib products require a 3-dose primary series with doses at ages 2, 4, and 6 months. Coverage for the full series, which includes the primary series and a booster dose, was based on receipt of ≥3 or ≥4 doses, depending on product type received. All Hib products require a booster dose at age 12–15 months.

§§ Coverage for rotavirus vaccine was based on ≥2 or ≥3 doses, depending on product type received (≥2 doses for Rotarix [RV1], licensed in April 2008, and ≥3 doses for RotaTeq [RV5], licensed in February 2006).

¶¶ Information on coverage with individual vaccines since the inception of NIS in 1994 through 2012 is available at http://www.cdc.gov/vaccines/stats-surv/nis/figures/2012_map.htm.

*** The 4:3:1:3*:3:1:4 vaccine series includes ≥4 doses of DTaP/diphtheria and tetanus toxoids vaccine/diphtheria and tetanus toxoids and pertussis vaccine, ≥3 doses of poliovirus vaccine, ≥1 doses of measles vaccine, ≥3 or ≥4 doses of Hib (depending on product type of vaccine), ≥3 doses of HepB, ≥1 doses of varicella vaccine, and ≥4 doses of PCV.

††† Poverty level uses income and family size to categorize households into 1) at or above the poverty level and 2) below the poverty level. Poverty level was based on 2011 U.S. Census poverty thresholds, available at http://www.census.gov/hhes/www/poverty/data/threshld.

§§§ Child’s race/ethnicity was reported by their parent or guardian. Children categorized in this report as white, black, Asian, American Indian/Alaska Native, or multiracial were identified as non-Hispanic by their parent or guardian. Children identified as multiracial had more than one race category selected. Persons identified as Hispanic might be of any race.

¶¶¶ Additional information about the Vaccines for Children program is available at http://www.cdc.gov/vaccines/programs/vfc/default.htm.

**** Additional information about WIC is available at http://www.fns.usda.gov/wic.

†††† Enrollment in the new Health Insurance Marketplace begins October 1, 2013. The Health Insurance Marketplace will offer individuals and small businesses a streamlined process to compare health plans, get answers to questions, find out if they are eligible for tax credits for private insurance or health programs like the Children’s Health Insurance Program (CHIP), and enroll in a health plan that meets their needs. Consumers can learn more about the Marketplace at http://www.healthcare.gov or the Spanish-language site http://www.cuidadodesalud.gov or by calling the 24-hour consumer call center at 1-800-318-2596. Hearing impaired callers using TTY/TDD technology can call 1-855-889-4325 for assistance.
